# Meta‐analysis and Consolidation of Farnesoid X Receptor Chromatin Immunoprecipitation Sequencing Data Across Different Species and Conditions

**DOI:** 10.1002/hep4.1749

**Published:** 2021-07-01

**Authors:** Emilian Jungwirth, Katrin Panzitt, Hanns‐Ulrich Marschall, Gerhard G. Thallinger, Martin Wagner

**Affiliations:** ^1^ Research Unit for Translational Nuclear Receptor Research Division of Gastroenterology and Hepatology Medical University Graz Graz Austria; ^2^ Institute of Biomedical Informatics Graz University of Technology Graz Austria; ^3^ OMICS Center Graz Graz Austria; ^4^ BioTechMed‐Graz Graz Austria; ^5^ Department of Molecular and Clinical Medicine/Wallenberg Laboratory Sahlgrenska Academy University of Gothenburg Gothenburg Sweden

## Abstract

Farnesoid X receptor (FXR) is a nuclear receptor that controls gene regulation of different metabolic pathways and represents an upcoming drug target for various liver diseases. Several data sets on genome‐wide FXR binding in different species and conditions exist. We have previously reported that these data sets are heterogeneous and do not cover the full spectrum of potential FXR binding sites. Here, we report the first meta‐analysis of all publicly available FXR chromatin immunoprecipitation sequencing (ChIP‐seq) data sets from mouse, rat, and human across different conditions using a newly generated analysis pipeline. All publicly available single data sets were biocurated in a standardized manner and compared on every relevant level from raw reads to affected functional pathways. Individual murine data sets were then virtually merged into a single unique “FXR binding atlas” spanning all potential binding sites across various conditions. Comparison of the single biocurated data sets showed that the overlap of FXR binding sites between different species is modest and ranges from 48% (mouse‐human) to 55% (mouse‐rat). Moreover, *in vivo* data among different species are more similar than human *in vivo* data compared to human *in vitro* data. The consolidated murine global FXR binding atlas virtually increases sequencing depth and allows recovering more and novel potential binding sites and signaling pathways that were missed in the individual data sets. The FXR binding atlas is publicly searchable (https://fxratlas.tugraz.at). *Conclusion:* Published single FXR ChIP‐seq data sets and large‐scale integrated omics data sets do not cover the full spectrum of FXR binding. Combining different individual data sets and creating an “FXR super‐binding atlas” enhances understanding of FXR signaling capacities across different conditions. This is important when considering the potential wide spectrum for drugs targeting FXR in liver diseases.

## Graphical abstract

We report the first meta‐analyses of all publically available FXR ChIP‐seq data and consolidate all single data sets into a new global FXR‐binding atlas using a newly developed analysis pipeline‐ We provide a comparison of FXR binding across different species and conditions including in vivo and in vitro settings‐ We consolidated all individual murine data sets and generated a virtually deeply sequenced new data set, which allows to identify putative binding sites, which were missed in the individual data sets‐ The consolidated data and all individual data across different species are easy accessible via our newly generated online search database.

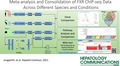

**Abbreviations**
bp: base pairs
ChIP: chromatin immunoprecipitation
ENCODE: Encyclopedia of DNA Elements
ER2: everted repeat 2
FXR: farnesoid X receptor
Gbp: gigabase pairs
GW4064: 3‐(2,6‐dichlorophenyl)‐4‐(3’‐carboxy‐2‐chlorostilben‐4‐yl)oxymethyl‐5‐isopropylisoxazole
H: human
hg: human genome
IgG: immunoglobulin G
IR1: inverted repeat 1
kbp: kilobase pairs
M: mouse
MET: metformin
mm: Mus musculus
NORM: normal
Nr0b2: nuclear receptor subfamily 0 group B member 2
OBES: obese
OCA: obeticholic acid
PCA: principal component analysis
R: rat
seq: sequencing
T+M: taurocholic acid plus metformin
TCA: taurocholic acid
TSS: transcription start site
VEH: vehicle

Farnesoid X receptor (FXR) is bile acid‐activated nuclear receptor and transcription factor that coordinates nutritional inputs and metabolic outputs of the liver and intestine.^(^ ^1^, ^2^ ^)^ In addition to transcriptional regulation of metabolic genes, FXR has anti‐inflammatory and antifibrotic properties. This array of established effects has put FXR in the spotlight as a novel therapeutic target for various metabolic liver diseases, including bile acid disorders and fatty liver disease.^(^ ^3^ ^)^ However, on a genomic level, FXR occupancy is not limited to these established metabolic pathways but spans a much wider range of largely unrecognized binding sites that might be occupied only under certain (patho)physiological conditions or after ligand activation.^(^ ^4^, ^5^ ^)^ Understanding precise genomic FXR binding and transactivation of genes is important to fully reconstruct FXR signaling, particularly when targeted by therapeutic drugs in diseased conditions.

Chromatin immunoprecipitation (ChIP) followed by next‐generation sequencing (ChIP‐seq) is a method to identify genome‐wide binding sites of a specific transcription factor and to gain information about transcriptional gene regulation, regulated pathways, and distinct binding motifs. Several FXR ChIP‐seq data sets for different species, conditions, and cell lines have been reported, and this has helped to extend understanding of the molecular and physiological actions of FXR.^(^ ^4^, ^5^, ^6^, ^7^, ^8^, ^9^, ^10^, ^11^ ^)^ Comparative ChIP‐seq studies on rodents and humans are largely lacking and divergent. One study compared FXR binding between primary human hepatocytes *in vitro* and mouse liver *in vivo* and found that the global FXR binding patterns were largely similar for mouse livers and human hepatocytes.^(^ ^9^ ^)^ Another study compared only the transcriptomic effects of FXR activation for mouse liver and human precision‐cut liver slices and found a surprisingly low number of overlapping genes in mouse and human.^(^ ^7^ ^)^ However, these individual studies show that the technical quality of single experiments and analyses have markedly evolved over the last decade and, importantly, that metabolic and tissue backgrounds as well as an underlying disease significantly determine FXR binding. Because the metabolic background (e.g., normal liver, fatty liver, inflamed or fibrotic liver) can change over a lifetime, FXR binding and effects of ligand activation may also change according to the metabolic/diseased background. An apprehension of global FXR binding possibilities, which takes the sum of information from the different individual experiments into consideration, is lacking. This information would be of particular importance for the nuclear receptor FXR, which is a promising drug target for liver diseases with various metabolic backgrounds.

A drawback of the single studies is that they are less accessible to bench biologists. Therefore, large‐scale databases, such as Transcriptomine,^(^ ^12^ ^)^ the follow‐up database Signaling Pathways Project,^(^ ^13^ ^)^ or the Chip Atlas,^(^ ^14^ ^)^ have been established that integrate several thousand cistromic, epigenomic, and transcriptomic data sets and make the data points online accessible and searchable for bench biologists. However, they only include a subset of the published FXR data sets. Moreover, pooling various data sets, which increases binding depth and enables extraction of novel information and noise reduction, is not possible in these large web resources.

Our aim was to answer the scientific question “What are the global binding sites of FXR that are accessible under all possible conditions?” To achieve this, we created a global FXR binding atlas independent of the experimental or metabolic condition. This global FXR binding atlas can be used for further extended downstream analyses of FXR signaling properties and is publicly searchable.

## Materials and Methods

### Data Sets

We searched public sources (National Center for Biotechnology Information [NCBI] Sequence Read Archive [SRA],^(^ ^15^ ^)^ Encyclopedia of DNA Elements [ENCODE],^(^ ^16^ ^)^ University of California, Santa Cruz [UCSC],^(^ ^17^ ^)^ The Signaling Pathways Project,^(^ ^13^ ^)^ Cistrome Data Browser,^(^ ^18^ ^)^ and the ChIP Atlas^(^ ^14^ ^)^) for available FXR ChIP‐seq data sets. By April 2020, five *in vivo* FXR ChIP‐seq data sets were available for mouse,^(^ ^4^, ^5^, ^6^, ^7^, ^8^ ^)^ one *in vivo* data set for rat,^(^ ^10^ ^)^ and one *in vitro* data set for primary human hepatocytes.^(^ ^9^ ^)^ We also had access to our own *in vivo* FXR ChIP‐seq data set from human liver tissue.^(^ ^11^ ^)^ The basic characteristics of the various data sets, including the study label, which consists of an abbreviation of the species (*H, M, R*) and the last author initials (*GG,* ^(^ ^4^ ^)^ *JK*,^(^ ^5^ ^)^ *JS*,^(^ ^10^ ^)^ *MW*,^(^ ^11^ ^)^ *PL*,^(^ ^8^ ^)^ *SK*,^(^ ^7^ ^)^ *TO* ^(^ ^6^ ^)^) from the respective data set, are shown in Table 1. Raw reads were available from NCBI SRA^(^ ^15^ ^)^ for all data sets except data sets *M_GG* and *M_TO*. For *M_GG*, only called peak tracks were available, which were shared by Grace Guo (GG).^(^ ^4^ ^)^ For *M_TO*, only mapped read tracks were available, which were provided by Chong et al.^(^ ^6^ ^)^ The eight individual data sets included different ChIP‐seq experiments (Table 1) so that a total number of 25 individual FXR ChIP‐seq samples were available. An overview of the individual samples from the various data sets can be found in Supporting Table S1. Individual sample names from the different data sets are a combination of the abbreviations for their species (S), condition (CCCC: NORM, normal; OBES, obese), treatment (TTT: BD (1,5,E), ligated bile duct for 1,5 or 14 days; GW4064, 3‐(2,6‐dichlorophenyl)‐4‐(3′‐carboxy‐2‐chlorostilben‐4‐yl)oxymethyl‐5‐isopropylisoxazole; MET, metformin; OCA, obeticholic acid; SH (1,5,E), 1,5 or 14 days after sham surgery; TCA, taurocholic acid; T+M, TCA+MET; VEH, vehicle), laboratory (LL), and identification (I) within the data set. This leads to a uniform naming format (S_CCCC_TTT_LL_I).

**Table 1:** Overview of publicly available FXR ChIP‐seq data sets

Data Set	Species	Tissue	Number of Samples	Experimental Condition	Antibody	Mapping Tool	Reference Genome	Peak Calling Tool	Control	FDR Cutoff	#Peaks	Peak to Gene Annotation	#Genes	*De Novo* Motif	Ref.
*M_TO*	Mouse [C57BL/6]	Liver	1	Normal	sc‐13063	ELAND	Reference genome by Ambry Genetics	MACS	IgG	0.05 (and *P* = 1e–5)	1.656	All FXR binding sites were assigned to nearest genes	1,038 (<20 kb distance to peak)	IR1	^(^ ^6^ ^)^
*M_GG*	Mouse	Liver and Intestine	2	Normal/ GW4064	H‐130x	Genome Analyzer Pipeline Software (Illumina)	No control	Region appeared more than 20 times	5,321 (liver)‐ 7,794 (intestine)	2 kb upstream of TSS	‐			No *de novo*	^(^ ^4^ ^)^
	[C57BL/6]														
*M_JK*	Mouse [BALB/c]	Liver	4	Normal diet/ high‐fat diet and DMSO/ GW4064	sc‐1204 and sc‐13063	CisGenome	mm9	CisGenome	IgG	0.001	5,272‐15,263	Gene within 10 kb distance to peak	1,566‐2,583 (unique genes of one group)	IR1	^(^ ^5^ ^)^
M_PL*	Mouse [C57BL/6]	Liver	4	Normal/TCA/MET/ TCA+Met	sc‐13063	Bowtie	mm9	MACS	No control	‐	>7,500	‐	‐	No *de novo*	^(^ ^8^ ^)^
M_SK*	Mouse [C57BL/6]	Liver	4	Normal/ OCA	sc‐13063	BWA	mm9	MACS1.4	Input	*P* = 1e–4	‐	10 kb upstream and downstream to gene	611 (regulated by FXR)	No *de novo*	^(^ ^7^ ^)^
*R_JS*	Rat	Liver	6	Sham/BDL	sc‐13063	BWA	rn5	MACS1.4	Input	*P* = 1e‐7	‐	10 kb upstream and 1 kb downstream from TSS	0‐3,908 (increased/decreased binding BDL to sham)	No *de novo*	^(^ ^10^ ^)^
	[CD‐IGS]														
*H_GG*	Human [PHH]	Liver	2	DMSO/GW4064	sc‐1204x and	Bowtie	hg19	MACS1.4 and Mali Salmon’s Peak Splitter	IgG	MACS: 0.1	2,759‐5,235	Peak within 10 kb upstream of gene	‐	IR1 (+ER2)	^(^ ^9^ ^)^
					sc‐13063x					Peak Splitter: *P* = 1e–5					
*H_MW*	Human	Liver	2	Normal/cholestasis	sc‐13063	Bowtie	hg19	MACS2	No control	0.05	6,601‐16,168	Overlap with gene or 1 kb upstream promotor	4,804‐8,555	IR1	^(^ ^11^ ^)^

Different data sets used different analysis tools and strategies. Data sets marked with * were used for the pooled data set.

Abbreviations: BDL, bile duct ligation; BWA, Burrows‐Wheeler Aligner; DMSO, dimethyl sulfoxide; hg, human genome; rn, *Rattus norvegicus*.

### ChIP‐seq Analysis

Raw read handling and read mapping information are provided in the Supporting Materials and Methods.

We used MACS2^(^ ^19^, ^20^ ^)^ (version 2.1.1) for FXR peak calling, applying various commonly used parameter combinations to evaluate effects on peak calling and determine the most reliable settings. These parameter settings included *Q*‐value cutoffs 0.01 or 0.05; using input DNA, immunoglobulin G (IgG), or no control sample; using a fixed or estimated fragment length; and two commonly used effective genome sizes for human samples (2.45 and 2.7 gigabase pairs [Gbp]). Peaks were further filtered using the ENCODE blacklist regions, which represent a comprehensive set of genomic regions with a high noise level in next‐generation sequencing data independent of cell line or experiment.^(^ ^21^ ^)^

### Peak to Gene Annotation and Pathway Analysis

Filtered peaks were annotated to UCSC known genes using the R package ChIP‐Seeker (version 1.18.0).^(^ ^22^ ^)^ Each gene was defined as potentially regulated by FXR if a peak overlapped with the gene or its promotor (the following promotor sizes were tested: 1 kilobase pair [kbp], 5 kbp, 10 kbp, and 20 kbp upstream from the transcription start site [TSS]). Genes annotated using a promotor size of 1 kbp were subjected to a REACTOME^(^ ^23^ ^)^ pathway analysis using the R package ReactomePA (version 1.28.0).^(^ ^24^ ^)^

### Data Set Comparison

We compared the data sets on the read and peak level based on the quality metrics proposed in ENCODE and other authoritative ChIP‐seq guidelines.^(^ ^25^, ^26^ ^)^ Similarity between the various peak calling results and their associated genes was determined using the Jaccard distance.^(^ ^27^ ^)^ We calculated the Jaccard distance based on the genes associated with the called peaks. The pairwise Jaccard distances were visualized with a heatmap. Genes were mapped to corresponding orthologues of other species to correctly estimate the similarity between different species. The Human Genome Organisation Gene Nomenclature Committee (HGNC) Comparison of Orthology Predictions (HCOP) database was used to find the orthologous genes for mouse and rat in humans.^(^ ^28^ ^)^ In the case of multiple orthologues for one gene, the one with the highest support was taken. Support was defined by the number of databases that contained the orthologue. Enrichment of pathways across samples is shown with dot plots created with ReactomePA.^(^ ^24^ ^)^ Additional pathway trees for each sample with enriched pathways were created to investigate the branch and subtree differences between the samples. An overview of this workflow is given in Supporting Fig. S1.

### Generation of a Pooled Data Set

For pooling, we selected the following eight individual mouse samples that were generated using the same FXR antibody (sc‐13063; Table 1): (*M_NORM_VEH_PL_1*, *M_NORM_TCA_PL_2*, *M_NORM_MET_PL_3*, *M_NORM_T+M_PL_4*, *M_NORM_OCA_SK_1*, *M_NORM_OCA_SK_2*, *M_NORM_VEH_SK_3*, *M_NORM_VEH_SK_4*). We combined the filtered and mapped reads of these samples into a new pooled data set, *M_POOL_ALL_MW_1*. For samples with more than 10 million deduplicated mapped reads, 500 subsamples were created by randomly selecting 10 million reads, which is the ENCODE guideline threshold for moderate ChIP‐seq samples (an exception was made for sample *M_NORM_OCA_SK_2*, which had 9.8 million deduplicated mapped reads). The subsamples of the individual samples were merged to create 500 technical mouse‐pool replicates. Peak calling was performed for each of those mouse‐pool replicates. Peaks were recentered around their summit and resized to 500 bp using DiffBind^(^ ^29^ ^)^ (version 2.10.0). Finally, only peaks present in at least 251 of the 500 mouse‐pool replicates (“majority rule”) were used as the final mouse‐pool peaks and all subsequent analyses. The technical mouse‐pool replicates were necessary to ensure that the signal for the recovered peaks (potential FXR binding sites) is conserved within the individual data sets.

### Motif Analysis

We performed a *de novo* motif analysis for the top 500 scoring peaks using the MEME suite (version 4.12.0.0).^(^ ^30^ ^)^ Sequences flanking the peak summit by 100 bp on either side were examined with default parameters. Additionally, a motif scan for the canonical inverted repeat 1 (IR1) (AGGTCAxTGACCT)^(^ ^31^ ^)^ and everted repeat 2 (ER2) (TGACCTxxAGGTCA)^(^ ^4^, ^32^ ^)^ FXR motifs was performed using the tool FIMO from the MEME suite. The scan was performed for the HOMER^(^ ^33^ ^)^ IR1 and the ER2^(^ ^32^ ^)^ FXR motifs across the narrow peaks and wider peak regions. Potential binding of FXR to any other motifs was not assessed. The narrow and wider peak region were defined as 250 bp and 1,000 bp upstream and downstream from the peak summit.

### Pathway and Gene Search Tool

For easy access to our results, we developed a web‐based search tool. The search tool gives access to the combined/pooled data set and allows comparing the individual samples for each potential FXR binding site across various treatment conditions as well as for binding strength. Binding strength is represented by the number of filtered deduplicated ChIPed reads within the potential binding site normalized to the total number of filtered deduplicated ChIPed reads. The mouse *Mus musculus* 10 (mm10) assembly was used as the reference.

## Results

Preliminary results, which report the heterogeneity of the single data sets and attempts to unify data sets from different resources, were presented at the thirteenth annual conference on Health Informatics Meets Digital Health in Vienna in 2019, and the extended meeting abstract has been reported in the conference proceedings.^(^ ^34^ ^)^ Inputs from the meeting led to our new analysis strategies and the development of the searchable FXR binding tool.

### Individual Data Sets

FXR ChIP‐seq data sets from three different species are publicly available; these are five for mice,^(^ ^4^, ^5^, ^6^, ^7^, ^8^ ^)^ one for rat,^(^ ^10^ ^)^ one for human primary hepatocytes,^(^ ^9^ ^)^ and one from human liver biopsy samples.^(^ ^11^ ^)^ Data sets included baseline FXR binding and FXR binding under pharmacologic treatment (i.e., FXR activation with different ligands) or diseased conditions (i.e., diet‐induced nonalcoholic fatty liver disease or cholestasis) (Table 1; Supporting Table S1). Baseline quality criteria among the different single data sets were heterogeneous (Supporting Table S3).

### Impact of Different Analysis Parameters on Results of Individual Data Sets

All data sets where raw reads were available (*M_JK*, *M_PL*, *M_SK*, *R_JS,* and *H_MW*) as well as data set *M_TO* were analyzed in a uniform manner using different variables to determine the optimal analysis strategy. The human data set *H_MW* also included both an input and IgG control sample, which was critical for analyzing the impact of different control samples in ChIP‐seq experiments. The significant impact of different parameter settings on ChIP‐seq fidelity has been reported in detail.^(^ ^35^ ^)^

#### Cutoff for *Q* Value and Fragment Size

**FIG. 1:**
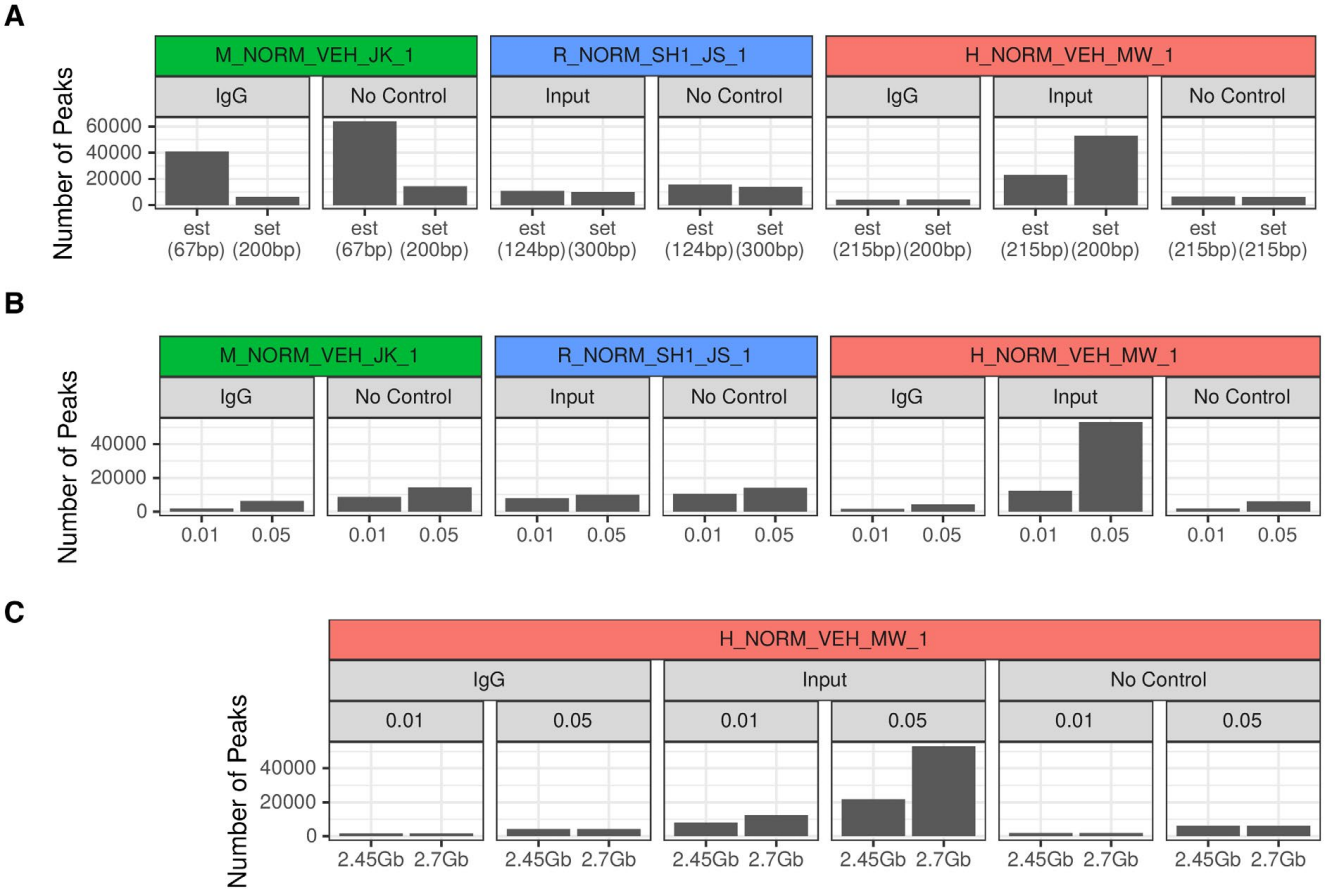
Impact of different analysis settings on the number of peaks. (A) Impact of the fragment size on number of peaks. Three representative samples are shown (mouse sample in green, rat sample in blue, and human sample in red). Background normalization was separately performed (dependent on availability) with input (DNA) control, no control, and IgG control. The *Q* value is set to 0.05 for all samples. Left bar represents number of peaks with estimated fragment size by MACS2 (est) and the right bar with fixed fragment size set to window size (set). If the fragment size estimation by MACS2 is close to the expected fragment size (window size), there are only minor differences between the two settings. (B) Impact of the *Q* value on number of peaks. Three representative samples are shown (mouse sample in green, rat sample in blue, and human sample in red). Background normalization was separately performed (dependent on availability) with input (DNA) control, no control, and IgG control. Fixed fragment size was set to window size. Left bar represents number of peaks with a *Q*‐value cutoff of 0.01 and the right bar with a cutoff of 0.05. Generally, the number of peaks increases with the increase of the *Q*‐value threshold. Depending on the control sample, the differences can be remarkable (e.g., input sample for the *H_NORM_VEH_MW_1* sample), suggesting introduction of bias. (C) Impact of effective genome size on number of peaks. Two different standard effective genome sizes are available for the human samples (2.45 Gb and 2.7 Gb). The *Q* value is set to 0.05 for all analyses. Depending on background normalization (Input) the number of called peaks markedly differ between the two genome sizes. Abbreviation: SH1, 1 day after sham surgery.

Changing the fragment size (Fig. 1A), which defines the minimum peak width, or *Q*‐value cutoffs (Fig. 1B) can have a substantial impact on the number of called peaks.^(^ ^35^ ^)^ This is best exemplified for the sample *M_NORM_VEH_JK_1*. Using an IgG control sample and estimating the fragment size by MACS2 resulted in 40,829 (Fig. 1A, green) and 5,189 (not shown) peaks for a *Q*‐value cutoff of 0.05 and 0.01, respectively. However, setting the fragment size to window size, as described in Lee et al.,^(^ ^5^ ^)^ results in only 6,320 (Fig. 1B, green) and 1,888 (Fig. 1B, green) peaks for a *Q*‐value cutoff of 0.05 and 0.01, respectively. In this scenario, the fragment size estimated by MACS2 is much smaller (67 bp) than the actual one (200 bp). Apparently, many small peaks with *Q* < 0.05, which are probably noise because they are shorter than the actual window size, lead to additionally called peaks.

#### Impact of Control Samples

Background normalization by a control sample should remove noise and false‐positive peaks and should result in a lower number of called peaks, which are more reliable.^(^ ^36^ ^)^ Background normalization is usually performed using an IgG or input DNA control, ideally from the same sample from which the ChIP has been performed. However, we found that the input and/or IgG control samples may also introduce additional noise rather than remove it. When using an IgG or input DNA control sample, additional peaks can be called, which then have a very low signal compared to their neighborhood. Using IgG, input DNA, or no control sample for the *H_NORM_VEH_MW_1* sample results in 4,301, 53,429, or 6,261 called peaks, respectively at a *Q*‐value cutoff of 0.05 (Fig. 1A,B, red). The significant impact on peak calling depending on the control sample has also been reported.^(^ ^35^ ^)^ Overall, this suggests that background normalization can potentially introduce further bias, particularly when comparing results derived from different normalization methods. With respect to the samples we analyzed in this study, some of the data sets did not include a control sample, some an input DNA control, and some an IgG control (Table 1). To ensure comparable results, we decided to analyze all samples without a control sample, as has been suggested.^(^ ^8^ ^)^ In this case MACS estimates the background from the ChIP‐seq sample itself. Because two different settings for effective genome size are commonly used for human samples (2.45 Gb and 2.7 Gb), we also determined the impact of the genome size on the number of called peaks. Depending on background normalization, the numbers of called peaks markedly differ between the two genome sizes (Fig. 1C).

The number of peaks called for in the different parameter settings in all samples is listed in Supporting Table S4. Based on the quality criteria and a comparison of the results with already established FXR targets, we considered the following parameter combination as the most reliable: (i) *Q* ≤ 0.05; (ii) no control sample; (iii) a fixed‐fragment length; and (iv) an effective human genome size of 2.7 Gbp (hg19) for the human samples. These parameters were used for all further analyses.

### Binding Motifs

FXR preferentially binds to an IR1 motif consisting of AGGTCAxTGACCT. This canonical IR1 motif was present in 3.9% (*M_OBES_VEH_JK_3*) to 55.8% (*M_NORM_VEH_TO_1*) of narrow peaks (defined as a 500‐bp‐wide region) and 20.2% (M_OBES_VEH_JK_3) to 64.5% (*M_NORM_VEH_TO_1*) in wider peak regions (defined as a 2,000‐bp‐wide region) for the different data sets. The ER2 motif was present in 5.4% (*M_OBES_VEH_JK_3*) to 39.0% (*M_NORM_VEH_TO_1*) of narrow peaks (defined as a 500‐bp‐wide region) and 30.7% (*M_OBES_VEH_JK_3*) to 61.6% (*M_NORM_VEH_TO_1*) in wider peak regions (defined as a 2,000‐bp‐wide region) for the different data sets (Supporting Table S5).

### Similarity of the Different Data Sets

**FIG. 2:**
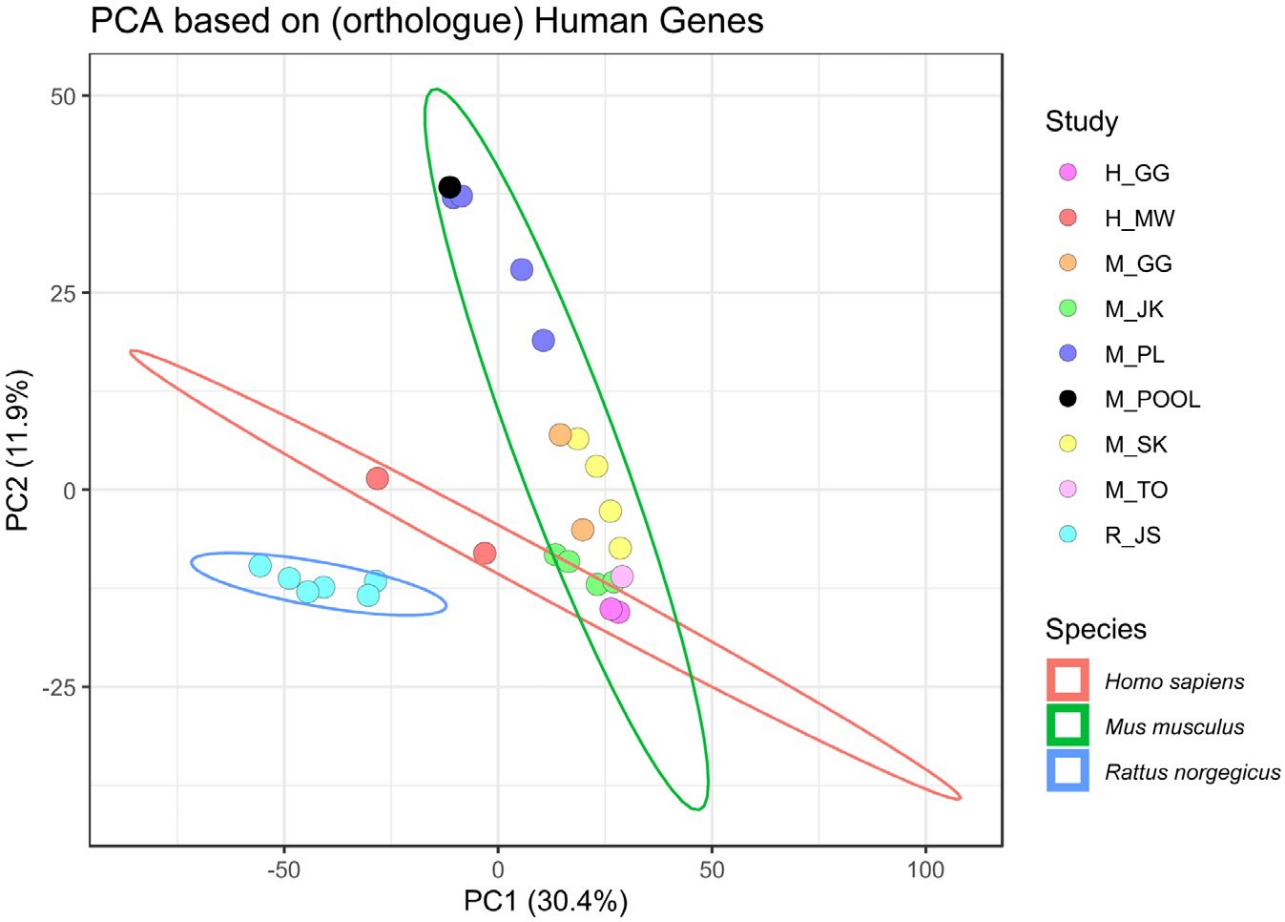
Similarity between data sets of different FXR ChIP‐seq studies. PCA based on the presence/absence of genes with potential FXR binding site for the samples. PCA shows clustering primarily according to data sets and secondarily to species. Rodent genes are mapped to their human orthologues to allow comparison of samples with different origins.

Principal component analysis (PCA) based on associated human orthologue genes shows that samples of the same data set cluster together rather than samples from the same condition/treatment from different data sets (Fig. 2). The impact of the data set appeared to be even stronger than the impact of the species. Of note, the human *in vivo* samples (*H_MW*) were closer to the rodent *in vivo* samples than to the human *in vitro* samples (*H_GG*). The Jaccard distance is an alternative measure for the dissimilarity of different data sets or samples. In line with the PCA, hierarchical clustering of the Jaccard distances based on the annotated genes also showed that samples preferentially cluster with samples of the same data set (Supporting Fig. S2).

### Pooling of Individual Data Sets and Characterization of the Combined Data Set

To virtually increase sequencing depth and thereby detect potentially new FXR binding sites as well as to determine the global FXR binding capacity across different conditions, we created a pooled sample from all individual mouse samples that had at least a moderate number of reads according to ENCODE standards (i.e., 10 million reads). This criterion was only met by eight samples from data sets *M_SK* and *M_PL* but included different experimental conditions. By pooling these samples on the read level and creating 500 random technical replicates from this mouse pool, a summation of the individual FXR signals was achieved. The summation of the FXR signals allows detection of weaker FXR binding sites, which are not detected in the individual samples because they are below the noise level. As the data sets are from different laboratories, only limited summation of technical noise was expected and relatively weak biological signals should be amplified. This analytical procedure combined with the strict filtering of the raw reads was expected to lead to a high‐quality virtually deep‐sequenced FXR ChIP‐seq data set.

**FIG. 3:**
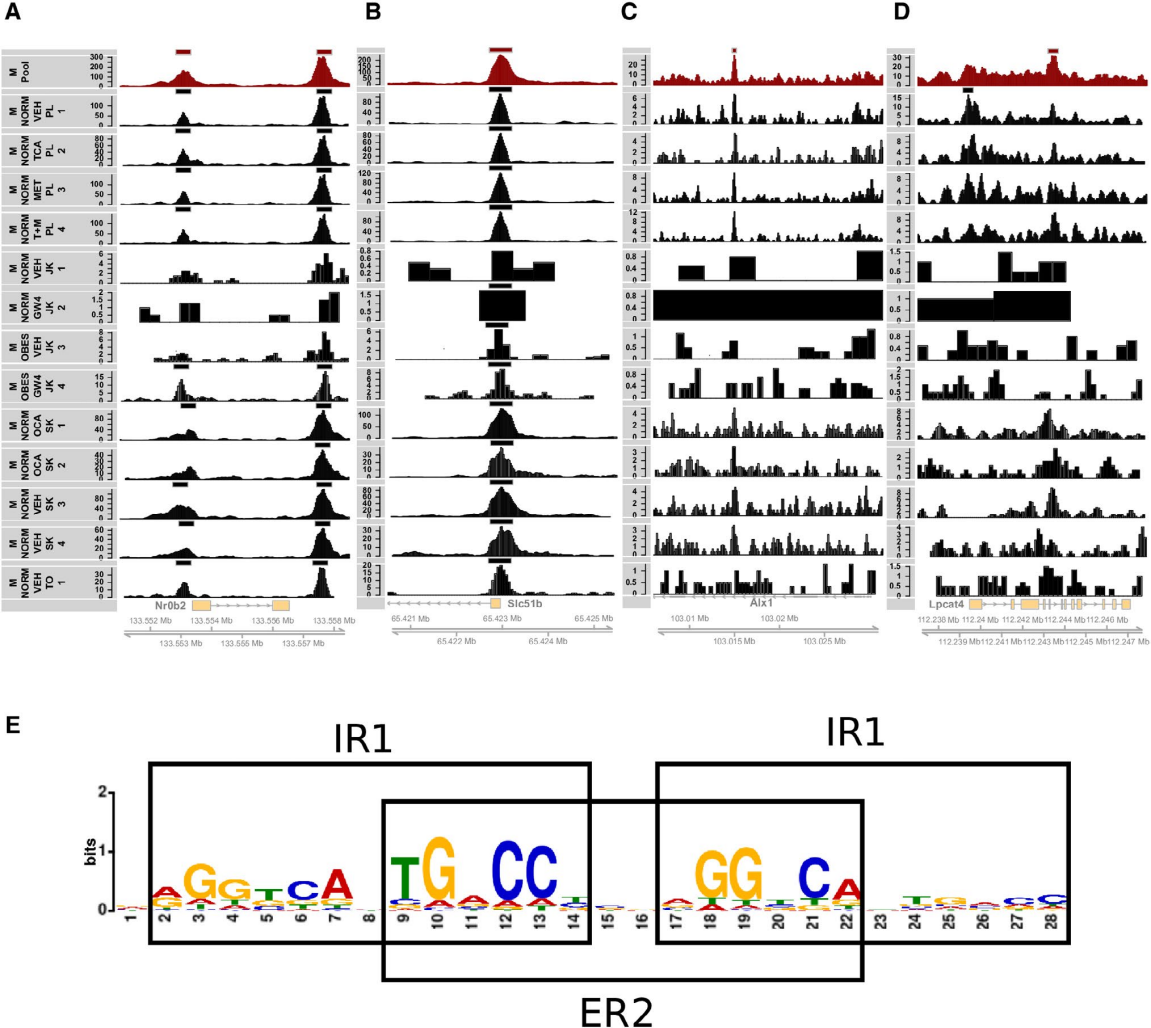
FXR binding peaks and motif of the pooled mouse data set. (A‐D) Examples of called peaks and ChIP read coverage. Established FXR targets (A) *Nr0b2* (*Shp*) and (B) *Slc51b* (*Ost‐β*). Two examples, (C) *Alx1* and (D) *Lpcat4*, are only called in the pooled data set (*M_POOL_ALL_MW_1*) but not in any individual sample. The called peak track of the pooled data sets (dark red) and the mean read coverage track of the pooled data set (*M_P*ool; dark red) are shown at the top. The called peak track of individual samples and the read coverage track (black) are presented below. (E) A *de novo* motif analysis reveals the canonical IR1 FXR response element and an additional adjacent IR1motif with a spacing of two bases; an ER2 motif is built between the two IR1 motifs. Overall, this forms a tetrameric motif with four half sites (AGGTCA) that was found in 288 of the top 500 peaks by MEME suite. Abbreviations: *Alx1*, ALX homeobox 1; *Lpcat4*, lysophosphatidylcholine acyltransferase; Mb, megabase; *Slc51b*, solute carrier family 51 subunit beta.

For the pooled data set, the number of called peaks was 13,599 and the number of associated genes 6,701. The pooled data set confirmed known FXR targets, such as nuclear receptor subfamily 0 group B member 2 (*Nr0b2*; alias *Shp*) and solute carrier family 51 subunit beta (*Slc51b*; alias *Ost‐β*) (Fig. 3A,B). Enhancement of weak signals after virtually increasing sequencing depth leads to the calling of novel peaks, such as peaks adjacent to ALX homeobox 1 (*Alx1*) and lysophosphatidylcholine acyltransferase 4 (*Lpcat4*) (Fig. 3C,D). The pooled data set revealed 2,557 new potential FXR binding sites that were not called in the individual mouse samples used for the pooled data set. However, 1,171 (46%) of these additional binding sites were called in at least one of the samples that were not included in the pooled data set. In addition, about 66% of the liver FXR ChIP‐seq genes from the *M_NORM_GW4_GG_1* data set, which was not included in the combined/pooled data set because only the peak tracks were available, were present in the combined data set. Furthermore, 23% of the *M_NORM_GW4_GG_1* genes, which were not present in any other individual mouse sample, were present in the pooled data set. This confirms the detection strength and validity of the pooling strategy. On the contrary, 5,640 binding sites (34% of all distinct binding sites in the individual data sets) were called only in the individual samples. A high proportion of these peaks were likely false positives that were filtered out during the pooling process.

In the pooled data set, the IR1 motif was present in 3,737 (27.4%) narrow peak and 5,613 (41.2%) wider peak regions (Supporting Table S5). The most prevalent motif identified by a *de novo* search within the top 500 peaks was the canonical FXR IR1 motif (AGGTCAxTGACCT). In line with a previous report,^(^ ^4^ ^)^ we also detected an additional nuclear receptor binding site in the immediate proximity of the canonical FXR IR1 motif. This additional site can correspond either to two IR1 motifs or to an ER2 motif with accompanying nuclear receptor half sites on both ends, forming a tetrameric motif (Fig. 3E). The putative tetrameric motif could be recovered in 28% (when using the default *P*‐value threshold of 1e–4) of all mouse‐pool FXR peaks.

**FIG. 4:**
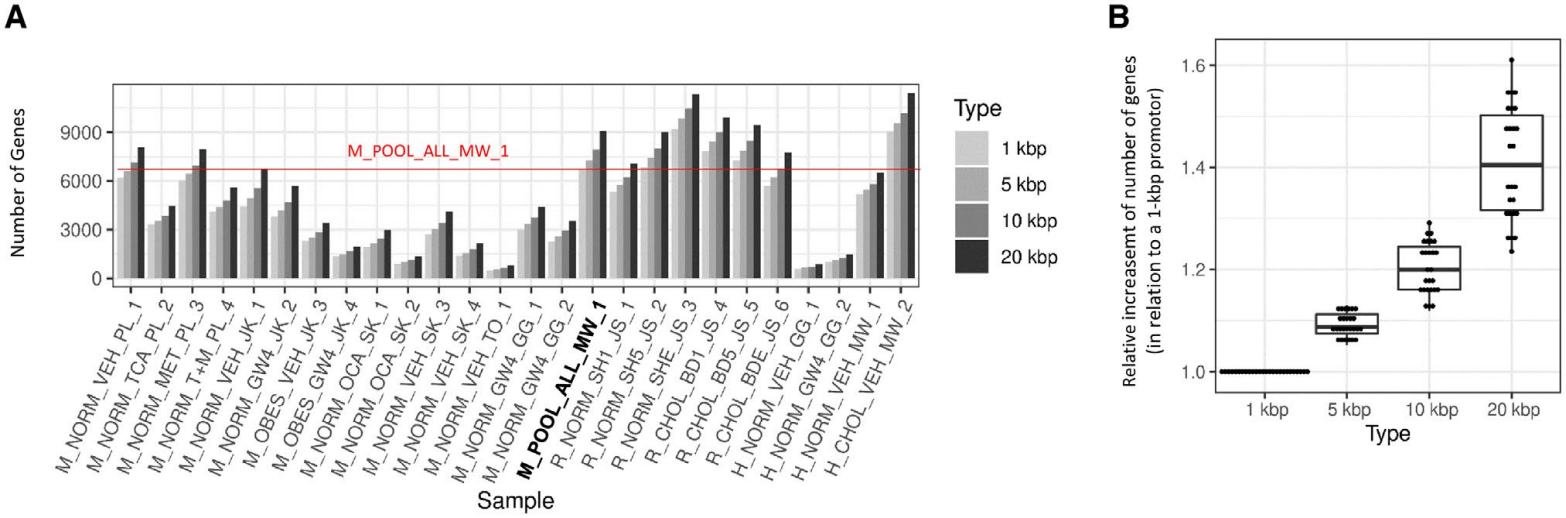
Impact of different promotor sizes on the total number of annotated genes per sample. (A) The number of annotated genes increases with the analyzed promotor size. The number of annotated genes in the combined/pooled data set is higher than the number of annotated genes in the individual mouse samples (red horizontal line marks the number of annotated genes in the mouse pool data set with a promotor size of 1 kbp upstream from the TSS). (B) The relative increase in the number of genes is small compared to the relative increase of the promotor size. Graphs show interquartile range (box), median (horizontal line), and outliers (whiskers). Abbreviations: BD (1,5,E), ligated bile duct for 1,5 or 14 days; CHOL, cholesterol; SH (1,5,E), 1,5 or 14 days after sham surgery.

Peaks were assigned to a gene if they overlapped with the gene body or the gene promotor. Depending on different promoter definitions, we could annotate 6,719, 7,297, or 7,959 genes for 1 kbp, 5 kbp, or 10 kbp upstream of the TSS, respectively (Fig. 4A; Supporting Table S6). The pooled data set recovered more genes than any individual mouse sample (Fig. 4A). On average, the increase of annotated genes was small compared to the increase of promotor size, e.g., increasing the promotor size from 1 kbp to 20 kbp increases the number of annotated genes on average by merely 40% (Fig. 4B).

**FIG. 5:**
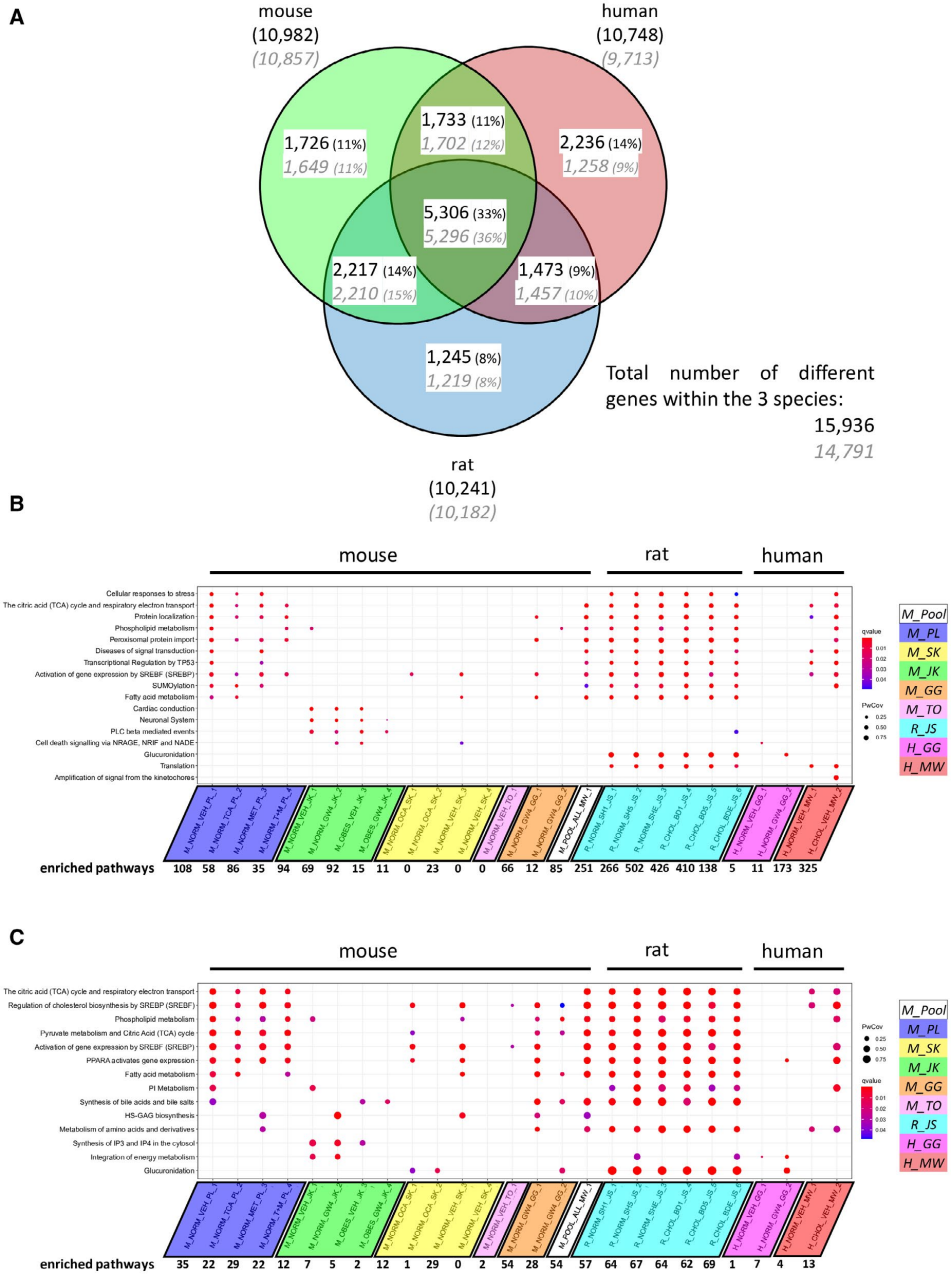
Gene and pathway comparison among different species. (A) Overlap of annotated genes between mouse, rat and human. Each circle represents all annotated genes (black) and protein coding genes (gray) across all samples within the respective species. Rodent genes are mapped to their human orthologues to allow comparison of different species. The highest overlap is observed between mouse and rat. (B,C) Dot plot of the (B) overall and (C) metabolic top enriched pathways. Rodent genes are mapped to their human orthologues. The orthologue genes are used for the enriched pathway analysis in the Reactome pathway database. Pathway analysis is limited to a gene set size between 10 and 500 and a *Q*‐value cutoff of 0.05. The total number of enriched pathways is provided at the bottom. Abbreviations: BD (1,5,E), ligated bile duct for 1,5 or 14 days; HS‐GAG, heparan sulfate/glycosaminoglycan; IP, inositol phosphate; NADE, p75 neurotrophin receptor‐associated cell deathexecutor; NRAGE, neurotrophin receptor–interacting melanoma‐associated antigen; NRIF, nuclear receptor interacting factor; PI, phosphatidylinositol; PLC, phospholipase C; PPARA, peroxisome proliferator activated receptor alpha; PwCov, Pathway Coverage ‐ ratio of genes from a pythway found in a sample; SH (1,5,E), 1,5 or 14 days after sham surgery; SREBF, sterol regulatory element‐binding transcription factor; SREBP, sterol regulatory element‐binding transcription protein; SUMO, small ubiquitin‐like modifier; TP63, tumor protein 63.

Comparison of rat and human data sets to the mouse pool data set according to the peak‐to‐gene profile showed that only 33% (5,309 of the overall 15,944) of annotated genes were present in at least one sample of each species. The highest overlap was between mouse and rat where 54.9% of annotated genes overlapped. The overlap between human and mouse was 47.9% and between human and rat 47.7% (Fig. 5A; note that these numbers represent the overlap between two species, whereas in the figure, numbers are based on the overlap of all three species).

Based on the annotated genes using a promotor size of 1 kbp, we performed a REACTOME^(^ ^23^ ^)^ pathway analysis (Fig. 5B; Supporting Table S7). Within the pooled data set, 83 significantly enriched pathways were found. Most of the significantly enriched pathways belonged to the “Metabolism” or “Signal Transduction” top layer pathways (Fig. 5B,C). Pathway trees for each sample are available in Supporting Figs. [Link], [Link], [Link], [Link], [Link], [Link], [Link], [Link], [Link], [Link], [Link], [Link], [Link], [Link], [Link], [Link], [Link], [Link], [Link], [Link], [Link], [Link], [Link]). The pathway analysis of the pooled data set revealed significantly enriched pathways, such as the “Notch‐HLH transcription” pathway, that are not present in any of the individual mouse data sets. Some of those additional pathways are also present in samples of the two other species; an example is the “Macroautophagy” pathway, which is present in human and rat samples (Table 2; Supporting Table S8). This demonstrates both a conservation of FXR dependency of that pathway across multiple species and validity of additional pathways identified by the combined data set.

**Table 2:** Number of pool genes/pathways not present in individual mouse samples. Genes and pathways that are only present in the combined (pool) mouse data set but not present in the individual mouse samples are compared to genes and pathways present in rat and human data sets. Most of the mouse genes and pathways overlap with the results of the rat and human samples

	Not in Mouse Samples	Overlap With	
		Rat	Human
Genes	180	109	91
Pathways	10	8	7

### Pathway and Gene Search Tool

**FIG. 6:**
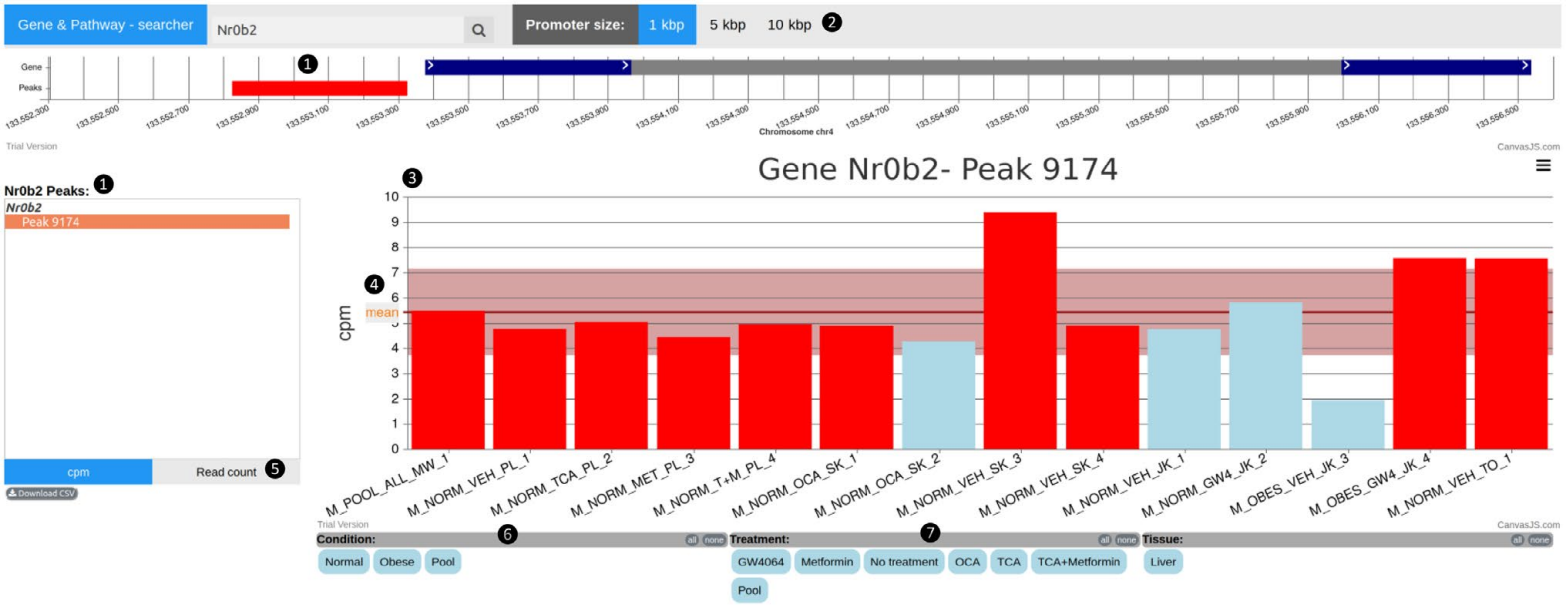
Online pathway and gene search tool. The screenshot shows the search results for the FXR target gene *Nr0b2* (*Shp*) with the selected promotor size 1 kb. There is a single peak (potential binding site) within the 1‐kbp upstream region of the gene (❶). Selectable promotor sizes are 1 kbp, 5 kbp, and 10 kbp (❷). For each sample, the number of reads within the peak normalized to the library size (cpm) is presented in the bar chart (❸). The mean cpm and SD for this peak is plotted as a horizontal red line and area, respectively (❹). As an alternative to the cpm, it is also possible to select the raw read count only (❺). Samples with a called peak are colored red, the others are colored blue. In the case of *Nr0b2*, the peak within the 1‐kb promotor is called for the pooled data set and nine of the 13 individual samples (red bars). In this given example, all 14 samples are displayed by default, but it is possible to deselect certain conditions (❻) and treatments (❼). Abbreviation: cpm, counts per million.

Based on our pooled FXR binding atlas, we developed an online search tool (https://fxratlas.tugraz.at) that allows searching for FXR binding sites within genes or pathways of interest. It also allows for easy comparison between different conditions and treatments (Fig. 6). The user can access data from the pool, from an individual sample, from all samples, or from a specific condition. Genes and their associated peaks (potential binding sites of the mouse pool *M_POOL_ALL_MW_1*) are displayed on a genome track. The number of reads within a peak normalized to the library size is presented in a bar chart to compare the occupancy between the samples for a given peak. A summary table for individual genes or entire pathways is available for download to further enhance the accessibility for the user.

## Discussion

Mapping FXR to its genomic binding loci allows a global prediction of functional pathways that are potentially affected by FXR binding. Mapping of FXR binding has been performed in several species and under various conditions with interesting results.^(^ ^4^, ^5^, ^6^, ^7^, ^8^, ^9^, ^10^, ^11^ ^)^ Here, we report the first meta‐analysis of all publicly available FXR ChIP‐seq data sets together with the combination of individual data sets; this provides a high‐quality global picture of all FXR binding sites across various conditions with detection of several new potentially FXR‐regulated genes and pathways.

Eight FXR ChIP‐seq data sets consisting of 25 single FXR ChIP‐seq experiments are publicly available from mouse, rat, and human^(^ ^4^, ^5^, ^6^, ^7^, ^8^, ^9^, ^10^, ^11^ ^)^ under different experimental conditions. These data sets were analyzed initially with considerably different parameter settings. As peak calling is highly sensitive to these settings, we defined a standardized set of parameters that we used in our re‐analysis. Most influential proved the choice of the control sample, which is generally underestimated in the studies. A low‐quality control sample can have significant impact on peak calling results even if the ChIP‐seq sample is of good quality. This influence of control samples on peak calling results was also reported in other studies.^(^ ^35^, ^37^ ^)^ Because a control sample was not available for all samples, we performed peak calling without control to ensure comparable results. With our standardized analysis pipeline, we could assess and compare all criteria and observed that the ENCODE thresholds are often not reached, which could influence subsequent peak calling as well.

An unexpected finding of our comparative analysis was that even after standardized analysis the individual samples clustered by study rather than by treatment or condition. This emphasizes the influence of laboratory procedures^(^ ^38^, ^39^ ^)^ and calls for extended quality control in the ChIP‐seq workflow. It is known that the antibodies used for ChIP account for a considerable proportion of the variability in the ChIP‐seq workflow^(^ ^40^ ^)^ and consequently could also affect our pooled data set. This is, however, not the case as the same antibody was used for all individual samples used for pooling.

Individual data sets often exhibit a sequencing depth that is too low to identify weak/rare binding sites, but deeper sequencing significantly increases experimental costs. In this study, we combined all suitable mouse reads to create a virtually deeply sequenced “FXR‐binding‐atlas” for a further robust downstream analysis of FXR signaling capacities. A potential bias within the combined data set might be the varying library size of the individual single data sets, which ranged from approximately 500,000 to 21,000,000 deduplicated reads. To overcome this potential bias, we aimed to create a pooled data set in which each individual sample contributes to the same extent to the overall result. We therefore randomly subsampled larger samples to a moderate number of reads (i.e., 10 million) and pooled these reads to create technical replicates. Using only consistent potential binding sites (which were called in the majority of the replicates) resulted in the pooled data set that was closest to an ideally merged data set with equal contribution of the individual samples. An important external validation was the high overlap with mouse samples, for which raw reads were not available and thus were not included in the combined data set. Additionally, about 75% and 70% of the annotated genes of the combined murine data set could be found as orthologues in at least one of the rat and human samples, respectively.

More genes were detected within the pooled data set than within the individual data sets alone, although we only used binding sites present in more than half of the technical replicates created for the pooled data set. The genes of those consistent binding sites revealed pathways that were not enriched in the individual samples. For example, the “macroautophagy” pathway is one of 10 pathways that are only enriched in the pooled mouse data set but not in the individual mouse samples. This is another important validation of our data because autophagy has been identified as a central FXR‐regulated pathway in several studies.^(^ ^11^, ^41^, ^42^ ^)^ Conversely, some peaks, genes, and pathways present in one or more individual mouse samples are not present in the pooled data set. An example is the testis‐specific and Y chromosome‐encoded murine pseudogene “*Tspy‐ps,*” which is not present in the pooled mouse data set although it is present in eight of the individual mouse samples. Signals for peaks that are not present in the pooled data set are not consistently found in the individual samples. This could be explained either by a weak signal that is only present under very specific conditions, which were only met in a single sample, or by peaks that were incorrectly called due to noise in the individual sample.

Comparing different species revealed that FXR binding and binding‐associated genes vary considerably. Although the well‐known and established genes and pathways of bile acid and cholesterol metabolism are shared among the different species, approximately 2,200, 1,700, and 1,200 genes are each unique to human, mouse, or rat, respectively. This is important to consider when rodent models are used to establish FXR as a drug target for various disease conditions. However, this observation is not specific to FXR but has been described for other nuclear receptors, such as peroxisome proliferator‐activated receptor gamma.^(^ ^43^ ^)^ Interestingly, the human *in vivo* liver samples were more similar to rodent *in vivo* samples than to *in vitro* human primary hepatocytes. Because we only had a single *in vitro* data set for comparison, it is not yet clear whether the differences are indeed true differences in binding between the *in vivo* and *in vitro* conditions or due to technically related issues. It has to be kept in mind that liver tissue is composed not only of hepatocytes but also of additional cells that harbor FXR, such as cholangiocytes, Kupffer cells, endothelial cells, and stellate cells. Potential differences in culturing conditions between *in vitro* and *in vivo* findings represent important confounders that must be considered when interpreting *in vitro* data.

*De novo* motif analysis of the pooled data set suggested a tetrameric motif. It consists of two canonical IR1 motifs separated by two bases; this forms an ER2 motif in the motif’s center. Overlapping IR1 and ER2 motifs have been reported for FXR.^(^ ^4^, ^9^ ^)^ However, it is currently not clear whether this tetrameric motif is an artefact caused by the overlap of the IR1 and ER2 motifs or represents a true response element for FXR.

A major drawback of the published genomic FXR data is that handling of these data sets and searching for specific binding sites requires bioinformatic expertise. Furthermore, from the eight published FXR data sets, only four are present in the large transcription factor web resources.^(^ ^13^, ^14^ ^)^ We, therefore, developed an easy to use, web‐based, FXR ChIP‐seq search tool comprising all currently available FXR data sets (https://fxratlas.tugraz.at) that allows (i) searching whether or not a specific gene of interest harbors FXR binding sites, (ii) comparing binding sites across different conditions, and (iii) searching for FXR binding‐enriched genes within biological pathways of interest.

There are limitations to our study and the pooled data set. First, as with any ChIP‐seq data, binding of a nuclear receptor does not necessarily result in altered transcription of the potentially regulated gene. A common strategy to overcome this drawback is the integration of ChIP‐seq data with either transcriptomic data or additional ChIP‐seq data that mark active transcription sites, such as RNA polymerase II (PolII) and/or distinct histone modifications.^(^ ^26^ ^)^ Because our pooled data set is the virtual consolidation of different single ChIP‐seq data sets, no integration with additional data sets was possible. Second, this study was intended as an *in silico* study only, and thus wet‐bench validation of novel FXR targets and pathways have to be performed in future investigations. Third, analysis has been undertaken in liver tissue, which is a mixture of different cell lineages. This study therefore gives no information of FXR binding within a specific cell lineage. Fourth, large‐scale databases that integrate thousands of data sets and that are publicly searchable exist^(^ ^12^, ^13^, ^14^, ^44^ ^)^ but have limited FXR coverage. In contrast, our study is focused on FXR and covers all publicly available FXR data sets and represents a unique resource because it combines very different data sets. While the large databases only reflect data from individual studies, we created a new data source based on our pooling approach with novel additional information on extensive FXR binding.

In summary, we generated a biocurated global FXR binding atlas that encompasses all potential FXR binding sites across various experimental conditions in mice. The FXR binding atlas is publicly available and will help wet‐bench biologists to specifically search for FXR‐regulated genes and pathways under various conditions.

## Supporting information

Fig S1

Fig S2

Fig S3

Fig S4

Fig S5

Fig S6

Fig S7

Fig S8

Fig S9

Fig S10

Fig S11

Fig S12

Fig S13

Fig S14

Fig S15

Fig S16

Fig S17

Fig S18

Fig S19

Fig S20

Fig S21

Fig S22

Fig S23

Fig S24

Fig S25

Table S1‐S8

Supplementary Material
