# Supplementary figures and images for: Meta‐analysis and Consolidation of Farnesoid X Receptor Chromatin Immunoprecipitation Sequencing Data Across Different Species and Conditions
Source: Hepatol Commun. 2021 Jul 1;5(10):1721–36. doi: 10.1002/hep4.1749 (PMC8485886; doi:10.1002/hep4.1749)

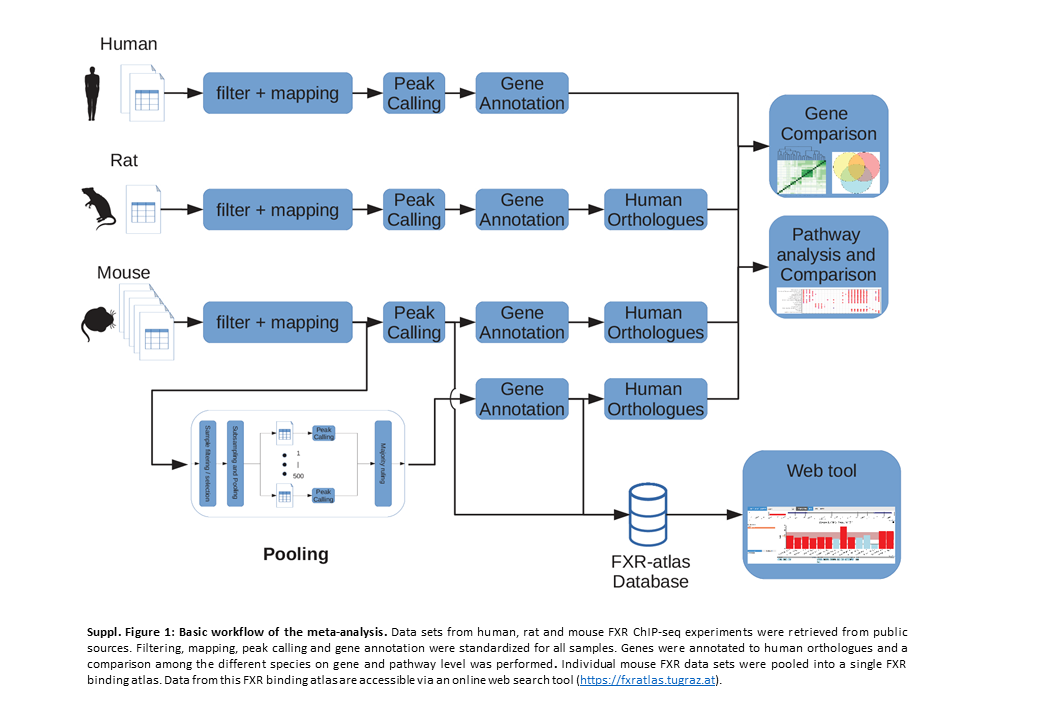

Supplement: Supplementary file 1 — Fig S1 [file HEP4-5-1721-s021.tif]

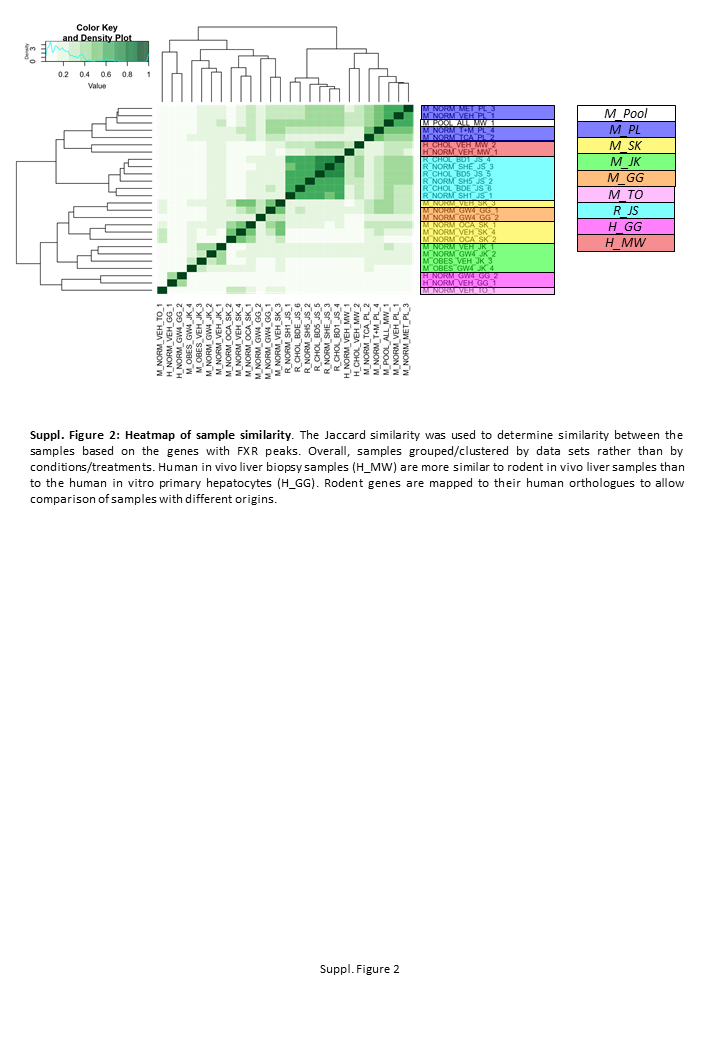

Supplement: Supplementary file 2 — Fig S2 [file HEP4-5-1721-s025.tif]
