# Supplementary material for: Meta‐analysis and Consolidation of Farnesoid X Receptor Chromatin Immunoprecipitation Sequencing Data Across Different Species and Conditions
Source: Hepatol Commun. 2021 Jul 1;5(10):1721–36. doi: 10.1002/hep4.1749 (PMC8485886; doi:10.1002/hep4.1749)
Supplement: Supplementary file 7 — Fig S7 [file HEP4-5-1721-s014.html]

diagonalNetwork


# Suppl. Figure 7: M\_NORM\_GW4\_GG\_1
