# Supplementary material for: Meta‐analysis and Consolidation of Farnesoid X Receptor Chromatin Immunoprecipitation Sequencing Data Across Different Species and Conditions
Source: Hepatol Commun. 2021 Jul 1;5(10):1721–36. doi: 10.1002/hep4.1749 (PMC8485886; doi:10.1002/hep4.1749)
Supplement: Supplementary file 18 — Fig S18 [file HEP4-5-1721-s018.html]

diagonalNetwork


# Suppl. Figure 18: M\_OBES\_VEH\_JK\_3
