# Supplementary material for: Meta‐analysis and Consolidation of Farnesoid X Receptor Chromatin Immunoprecipitation Sequencing Data Across Different Species and Conditions
Source: Hepatol Commun. 2021 Jul 1;5(10):1721–36. doi: 10.1002/hep4.1749 (PMC8485886; doi:10.1002/hep4.1749)
Supplement: Supplementary file 27 — Supplementary Material [file HEP4-5-1721-s011.docx]

**Suppl. Material and Methods:**

***ChIP-seq analysis***

Raw read handling: The datasets *M_JK*, *M_PL*, *M_SK*, *R_JS* and *H_MW* comprise single-end (SE) reads, the dataset (*H_GG*) paired-end reads. We used Trimmomatic [[1](#_ENREF_1)] (version 0.36.5) to trim and filter overrepresented sequences such as Illumina adapters. In addition to the ILLUMACLIP parameter, a SLIDINGWINDOW of 4 bases with an average quality of 28 and a minimum length of 80% of the raw read length were specified to ensure a high read quality. FastQC (<https://www.bioinformatics.babraham.ac.uk/projects/fastqc/>) was used to confirm the quality.

Read mapping: We mapped filtered reads to the mouse genome version mm10 (<https://www.ncbi.nlm.nih.gov/assembly/GCF_000001635.20/>), the rat genome version rn6 (<https://www.ncbi.nlm.nih.gov/assembly/GCF_000001895.5/>) and the human genome version hg19 (<https://www.ncbi.nlm.nih.gov/assembly/GCF_000001405.13/>) using Bowtie 2 [[2](#_ENREF_2), [3](#_ENREF_3)] (version 2.3.4.2) with default parameters. All murine studies were performed with mice from a C57BL/6 genetic background except *M_JK*, which used BALB/c mice (Table 1). Therefore, *M_JK* data was additionally mapped to the BALB/c genome (<https://www.ebi.ac.uk/ena/browser/view/GCA_001632525.1>) and the mapping statistics were compared. Based on this comparison and consistent with the original *M_JK* publication [[4](#_ENREF_4)], the mapping results from the mm10 were used.

**References**

[1] Bolger AM, Lohse M, Usadel B. Trimmomatic: a flexible trimmer for Illumina sequence data. Bioinformatics 2014;30:2114-2120.

[2] Langmead B, Trapnell C, Pop M, Salzberg SL. Ultrafast and memory-efficient alignment of short DNA sequences to the human genome. Genome biology 2009;10:R25.

[3] Langmead B, Salzberg SL. Fast gapped-read alignment with Bowtie 2. Nature methods 2012;9:357-359.

[4] Lee J, Seok S, Yu P, Kim K, Smith Z, Rivas-Astroza M, et al. Genomic analysis of hepatic farnesoid X receptor binding sites reveals altered binding in obesity and direct gene repression by farnesoid X receptor in mice. Hepatology 2012;56:108-117.
